# Who Is Spreading Avian Influenza in the Moving Duck Flock Farming Network of Indonesia?

**DOI:** 10.1371/journal.pone.0152123

**Published:** 2016-03-28

**Authors:** Joerg Henning, Dirk U. Pfeiffer, Mark Stevenson, Didik Yulianto, Walujo Priyono, Joanne Meers

**Affiliations:** 1 School of Veterinary Science, University of Queensland, Gatton, Queensland, Australia; 2 Royal Veterinary College, University of London, London, United Kingdom; 3 Faculty of Veterinary and Agricultural Sciences, The University of Melbourne, Parkville Victoria, Australia; 4 Disease Investigation Centre, Wates, Indonesia; St. Jude Children's Research Hospital, UNITED STATES

## Abstract

Duck populations are considered to be a reservoir of Highly pathogenic avian influenza (HPAI) virus H5N1 in some agricultural production systems, as they are able to shed the virus for several days without clinical signs. Countries endemically affected with HPAI in Asia are characterised by production systems where ducks are fed on post-harvest spilled rice. During this scavenging process it is common for ducks to come into contact with other duck flocks or wild birds, thereby providing opportunities for virus spread. Effective risk management for HPAI has been significantly compromised by a limited understanding of management of moving duck flocks in these countries, despite of a small number of recent investigations. Here, for the first time, we described the management of moving duck flocks and the structure of the moving duck flock network in quantitative terms so that factors influencing the risk of HPAIV transmission can be identified. By following moving duck flock farmers over a period of 6 months in Java, Indonesia, we were able to describe the movement of flocks and to characterise the network of various types of actors associated with the production system. We used these data to estimate the basic reproductive number for HPAI virus spread. Our results suggest that focussing HPAI prevention measures on duck flocks alone will not be sufficient. Instead, the role of transporters of moving duck flocks, hatcheries and rice paddy owners, in the spread of the HPAI virus needs to be recognised.

## Introduction

Highly pathogenic avian influenza (HPAI) H5N1 has remained endemic since at least 2004 in parts of Asia such as Indonesia and Vietnam, where 57% of South East Asia’s ducks are raised and where a large number of all human cases have been reported [1–2]. Duck populations are considered to be a reservoir of HPAI virus (HPAIV) H5N1 in endemically affected Asian countries as they are able to shed the virus for several days without clinical signs [3–4]. Their management is characterised by localised production systems where ducks are fed on post-harvest spilled rice and during scavenging ducks can come into contact with other duck flocks or wild birds, thereby providing opportunities for virus spread [5–6]. Despite risk management efforts targeted at the flock level (such as vaccination and partial confinement) a limited understanding of duck management systems in these countries has compromised the success of control efforts. In recent investigations two distinct management systems were identified in Indonesia and Vietnam; firstly a ‘stationary’ duck management system in which ducks are grazed around the household or village vicinity and returned over night to the village household and secondly a ‘moving’ duck management system, in which duck flocks are moved between areas of recent rice harvests and only kept in confinement overnight close to the day-time scavenging locations [7–8]. Only anecdotal evidence with respect to the latter exists, mainly because collection of detailed information about the management of these flocks is difficult due to the ducks being moved frequently. The objectives of this study were to study the movement and the management of moving duck flocks so that factors influencing the risk of HPAIV transmission can be identified. Furthermore, we aimed to investigate a network of professional contacts between people associated with the moving duck production system based on indirect links between them and duck flocks and highlight their potential impact on the spread of HPAIV. The data collection was conducted in Indonesia the country with the highest number of human cases and poultry cases of avian influenza [9–10].

## Materials and Methods

### Moving duck flocks

As no information on moving duck flocks existed in the study region, this information was compiled through rural appraisals [11] with village head men, local officials and farmers in four districts of Central Java. As differences in management practices and network structures in regions with varying water and rice paddy density were anticipated, two districts in coastal areas (Pemalang, Batang) and two districts in central inland areas (Klaten, Purworejo) were selected. A total of 48 moving ducks flock owners were identified and 27 moving duck flocks were then randomly selected and monitored monthly between November 2008 and April 2009. Moving duck flocks were visited by veterinarians from the Disease Investigation Centre (DIC) in Wates, Yogjakarta, district veterinarian and veterinary technicians. At each visit, latitude and longitude coordinates at the point of scavenging of duck flocks were obtained using a global positioning system (GPS) device. A baseline questionnaire was used to describe the practice of moving duck management at the start of the study and monthly questionnaires were used to provide details on transportation and the health status of birds’ between visits (S1 File).

None of the moving duck flocks followed were vaccinated against HPAI. Ten birds per flock were individually identified with wing bands and blood samples, cloacal and oropharyngeal swabs were collected from these birds at each visit. Serum samples were tested for HPAIV antibodies using the haemagglutination inhibitions test [12] and swabs were tested for the presence of HPAIV RNA using real-time Polymerase Chain Reaction [13]. All data collection for this study was conducted in accordance with the accepted guidelines of the DIC Wates, Indonesia and was approved by Directorate General of Livestock Services, Indonesia. As all activities were carried out as part of routine veterinary surveillance activities by the DIC, no additional animal or human ethics approval was required for this research.

The geographical coordinates of moving duck locations were plotted using ArcGIS 10.0. Flock movement distances were calculated as the Euclidean distance between the point locations recorded for two sequential scavenging locations. A journey was defined as a completed movement from a starting location to an end location, with ducks being released and allowed to scavenge at the end location. The cumulative distance travelled and the duration of scavenging at each location was calculated for the total study period of 6 months and for each month in which a journey commenced.

Moving duck flocks owners also identified all professionals involved in the moving duck management (referred in this manuscript as ‘actors’) over the 6-month study period. Actors included 1) transporters of moving duck flocks, who load duck flocks on their transport vehicles and move them between scavenging locations, 2) hatcheries, who purchase eggs and sell ducklings back to moving duck flock owners and 3) rice paddy owners, who make their rice paddies available to moving duck flocks for scavenging. Actors involved in the duck production/management were then visited by the survey teams and information on their professional activities involving moving ducks were collected using a questionnaire. GPS coordinates were obtained for each relevant location (e.g. site of the hatchery, of the rice paddy and of the transport provider vehicle storage place).

### Network development and analysis

Network analysis was used to explore the extent of the contacts between moving duck farmers and actors in order to understand the potential risk for HPAIV spread in the moving duck networks [14].

Networks between moving duck farmers and the three types of actors involved in the moving duck flock production system were cross-tabulated in a 2-mode matrix for each district. These networks consisted of nodes, which are the moving duck farmers and the actors involved in the duck management. Links or ties between the nodes represent relationships between moving duck farmers and the actors, i.e. if a particular moving duck farmer has used a specific actor. Each moving duck farmer may have had ties to one or more actors, whilst each actor might have had ties to many moving duck farmers. To be able to calculate social network analysis metrics, this 2-mode matrix was converted into a one mode matrix with the moving duck farmer providing the link between the actors. This approach was useful for ascertaining how connected actors are by some mutual affiliation with moving duck farmers. As the ties between moving duck farmers and the actors were assumed to occur in both directions, the binary ties in the networks were assumed to be undirected.

The following descriptive network measures were then calculated for each district: size of the network represented by the number of nodes or actors involved in the moving duck management, mean centrality measures (degree, betweeness and closeness) summarizing the position of the nodes or actors in the moving duck network and finally network centralisation reflecting the variability in the centrality of individual nodes or actors and thereby providing an idea of the overall cohesion of the moving duck network.

#### Degree

In an undirected binary graph, degree centrality measures the extent to which a node connects to all other nodes in a social network, i.e. the number of ties of a particular node [15]. Degree in the moving duck network therefore represents the number of actors that an actor is connected to via the moving duck flock farmers. In general, the greater an actor’s degree the more potential influence an actor has on the network and vice versa. For example, in a network in which HPAIV is present, an actor that has more connections can spread HPAIV more quickly, and will also be more likely to be infected. Therefore a high degree centrality implies a higher risk of either acquiring or spreading disease. As risk of HPAIV transmission is based on ties between different categories of actors, the social networks described here combine different HPAIV transmission pathways.

#### Closeness

Closeness represents the distance from one node to all others. Higher values are indicative of greater centrality, indicating that actors can be reached ‘quicker’ along network paths assuming that HPAIV transmission occurs faster.

#### Betweeness

Betweeness is the frequency with which a node falls between pairs of other nodes on the geodesic path connecting them. The betweenness of a node gives an idea of the amount of flow within the network that is ‘controlled’ by this particular node, assuming that the flow occurs along shortest paths. For HPAIV in the moving duck network, the closer an actor is to the middle of the network the larger are the number of pathways among actors in which it lies (i.e. the higher the betweenness).

#### Normalisation of centrality measures

Actor degree centrality measures do not only reflect each node's connectivity to other nodes but also depend on the size of the network; that is, the larger the network, the higher the maximum possible degree centrality value [16]. To eliminate the effect of variation in network size we standardized the measures of degree centrality [16]. Thus the differences between the largest centrality value in the network and all other observed values, was divided by the maximum possible sum of differences for a network of the same size [16].

All three measures of centrality were normalized for each of the four districts. Normalized degree was log-transformed and compared between the different actors and between districts using a one-way analysis of variance.

#### Network structure and connectedness

Embedded within a network there are often groups of nodes who interact with each other. These subgroups can be described by components and cutpoints. Components represent the sets of nodes that are connected to each other, while cutpoints represent the nodes whose deletion increases the number of components in the network.

Hence in a component every actor can reach every other by some path (no matter how long). In larger duck management networks numerous components might exist, each of these components representing a different group of actors between which HPAIV could potentially be spreading. Removing cutpoints from a network of actors will result in fragmentation of the network and may be important for targeting interventions to limit HPAIV spread.

Fragmentation, the proportion of pairs of actors that are unreachable was also calculated. It is expressed as an index between 0 and 1 and describes how disconnected the moving duck networks are. Furthermore density, the number of links present in the network, was calculated. It represents the proportion of all possible links between actors that are actually present in the network—values closer to 1 indicate that more possible links in a network are used. Finally, the overall clustering coefficient as the mean of the clustering coefficients of all the actors was derived. In a network with high clustering, two actors each linked to a third node have a high probability of being directly linked. High clustering indicates that on average, focal actors are surrounded by other actors that are well connected to each other [16]. The "weighted" version of the clustering coefficient gives weight to the neighborhood densities proportional to their size; that is, actors with larger neighborhoods get more weight in computing the average density [17].

All network calculations were performed using UCINET 6.465 (Borgatti, S.P., Everett, M.G., Freeman, L.C. Ucinet for Windows: Software for Social Network Analysis. Harvard, MA, Analytic Technologies. 2002). Network maps were produced in in R 3.0.2. (R Foundation for Statistical Computing) using the igraph package [18]. In ArcGIS 10.0 (ESRI. ArcGIS Desktop: Release 10. Environmental Systems Research Institute, Redlands, CA, USA. 2011) we calculated the K-function to quantify spatial autocorrelation among actors for each individual district. We used 999 permutations to generate a confidence envelope and accounted for locations near the study area boundary (a minimum enclosing rectangle was applied as study area polygon) by simulating outer boundary values.

### Calculation of the basic reproductive number

As these social networks based on ties between actors represent potential HPAI virus transmission routes, we used the network characteristics to calculate the basic reproductive rate (R_0_) as the average number of infected flocks resulting from transmission of virus after a single infected flock has entered a susceptible population.

The basic reproductive rate is the average number of cases resulting from transmission after a single case has entered a susceptible population [19]. If R_0_ is greater than 1, then epidemic spread is likely. R_0_ is calculated as the product between transmission probability (β), the average number of contacts (κ) and duration of infectivity (D) under the assumption of homogenous mixing, i.e. all members of the population, on average, have the same number of contacts [20], thus R_0_ = β × κ × D. The number of contacts (κ) is represented by the mean normalised degree derived from the social network analysis of the moving duck networks. As the rate of spread of an infectious agent can be influenced by the network structure, we used the normalised mean network degree centrality to calculate R_0_ [21–22].

Duration of infectivity (D) and the transmission probability (β) were derived from published literature. Henaux et al (2011) [23] indicated that the median infectious periods for LPAIV infection of ducks is 10–11.5 days and for HPAIV infection of ducks is 5 days—the latter figure was used in our analysis. Transmission probability in the social network described here is based on indirect contacts between actors. Thus transmission probabilities are based on contacts that occur, when a vehicle is used for transporting and delivering ducks, ducklings or eggs. Few estimates of transmission probability have been published. We assumed that the transmission probability (β) for the transport of ducks is similar to vehicles delivering feed to chicken farms during the HPAI outbreaks in the Netherlands (β = 0.0414) [24].

Most networks are heterogenous and this can be characterised by the degree of heterogeneity specified by the coefficient of variation (CV) [21]. The CV is the ratio of the standard deviation of normalised degree and the mean normalised degree. It was incorporated into the calculation of R_0_: R_0_ = β × κ × D × (1 + [CV]^2^). We calculated R_0_ for the four heterogeneous social networks of moving duck management of Central Java.

### Ethical considerations

All data collection for this study was conducted in accordance with the accepted guidelines of the DIC Wates, Indonesia and was approved by Directorate General of Livestock Services, Indonesia. Not all farmers were literate enough to understand a written consent form, so to be consistent, it was decided to obtain a verbal consent from all participants involved in the survey. Verbal consent was noted on the questionnaire used for the farmer interview. A copy of the questionnaire is provided as supplementary file (S1 File). No identifying information was collected from farmers. The survey responses used in this study were anonymized by the DIC veterinarians, who are the co-authors of this scientific paper. Because all activities were carried out as part of routine veterinary surveillance and disease investigation activities by the DIC, no additional animal or human ethics approval was required for this research. Interviews and sample collection were performed in accordance with the relevant guidelines and regulations of the Directorate General of Livestock Services, Indonesia. The Directorate General of Livestock Services, Indonesia could respond to specific questions on the conduct of the farmer survey if such questions arise. Sampling of ducks was conducted by experienced veterinarians and veterinary technicians from the DIC Wates using standardised sampling techniques. Their excellent knowledge and skills in sample collection minimised any possible suffering of ducks during the sampling process.

## Results

### Purpose of keeping moving duck flocks

All 27 flock owners indicated that moving duck farming was their most important income-generating activity. A total of 44% (N = 12) of flock owners specified that they received additional important income from rice production, 7% (N = 2) from pigs and 4% (N = 1) from chickens, other animals or other sources. For a total of 93% (N = 25) of flock owners, the sale of eggs (for human consumption) was the main purpose of keeping moving ducks, followed by home consumption of eggs (44%, N = 12), sale of fertile eggs for consumption (22%, N = 6) and sale of mature layer ducks. Sale of male ducks for slaughter (15%, N = 4) and sale of fertile eggs for hatching (15%, N = 4) were of lesser importance, while home consumption of duck meat (7%, N = 2) and sale of meat ducks were least important (4%, N = 1).

### Management and movement of duck flocks

**Fig 1:**
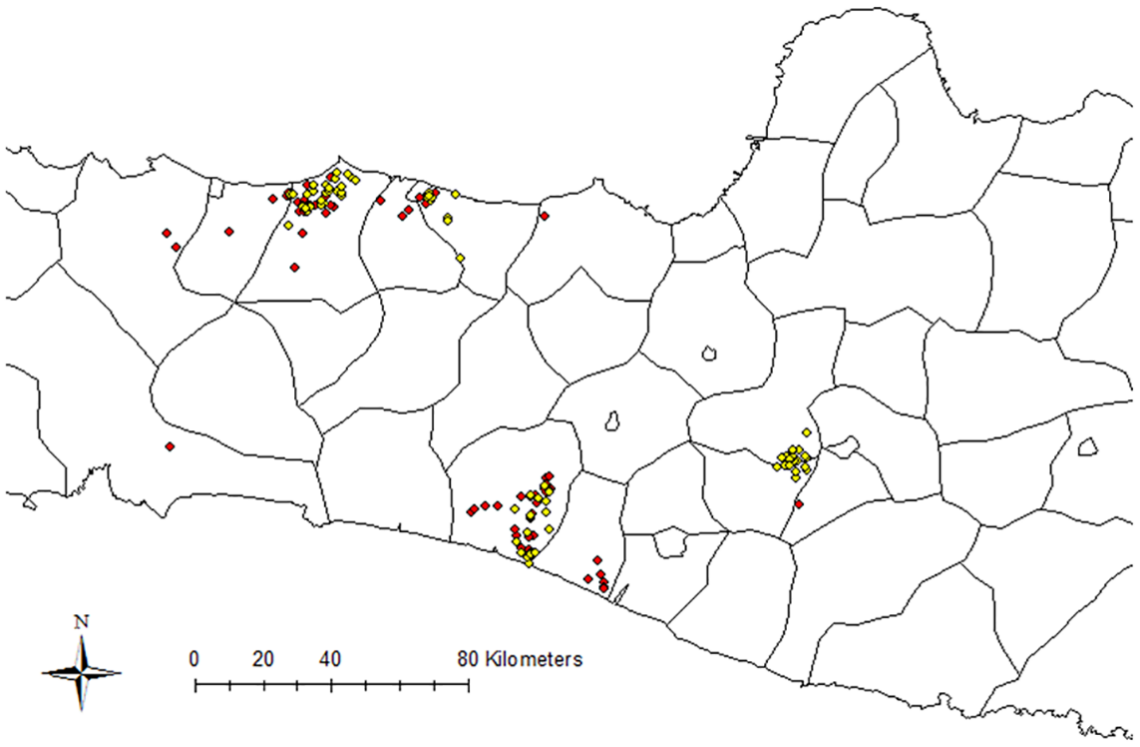
Locations of scavenging locations of moving duck flocks (in red) and of professionals (actors) involved in the moving duck management (in yellow) in four districts of Central Java, Indonesia. Locations of actors include the sites of hatcheries, the sites of the rice fields provided by rice farmers to moving duck flocks and the sites of the main storage places of vehicles used for transporting moving ducks). The map was generated in ArcGIS 10.0 (ESRI Inc., Redlands, CA, USA).

We collected detailed spatial information on movements of 27 moving duck flocks in four districts of Central Java, Indonesia over a period of six months (Fig 1).

**Fig 2:**
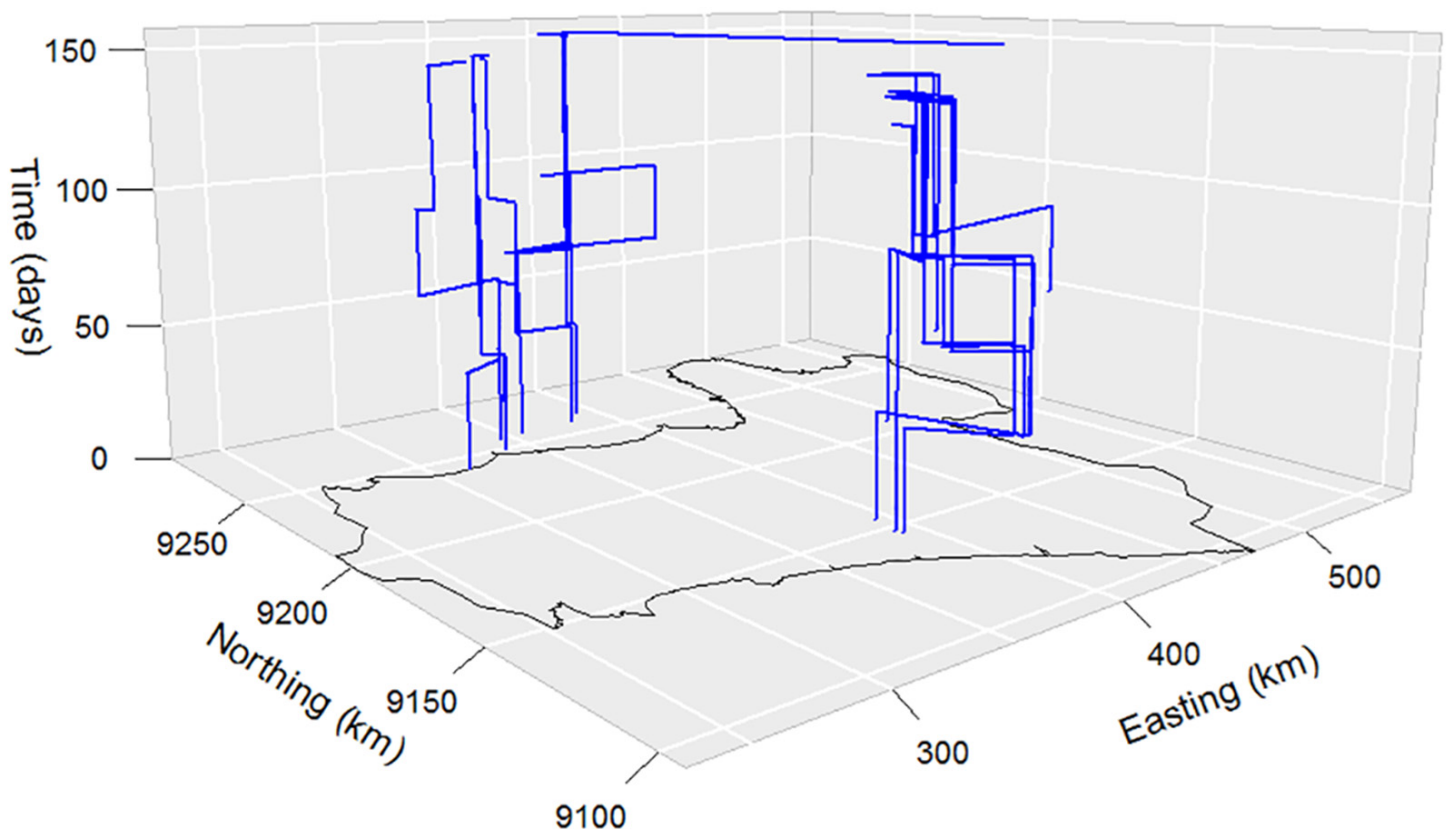
Three dimensional line plot showing the movement patterns of moving duck flocks on the Island of Java, Central Indonesia, over the 150-day study period. Horizontal lines represent distance travelled in kilometres, vertical lines represent the duration for which a flock was present at a specific location in days. The map was generated using GRASS 6.4.4 (http://grass.osgeo.org/).

Seventy-eight different scavenging locations were identified (locations less than 250 metres apart were assumed to refer to the same scavenging location). Over the 6 month monitoring period, an average of 2.9 scavenging locations were used per individual moving duck flock. There were regional differences in travel patterns (Fig 2, Table 1) with the average distance travelled during any journey being 23 km (median 13.8 km) and a large variation between journeys (standard deviation, SD = 26 km) (Table 2). The mean duration at any scavenging location was 59 days (median 48 days) with a SD of 37 days. The longest period at a single location was 169 days and the shortest period 26 days.

**Table 1:** Descriptive statistics on travel distance and duration of time spent per district per month for moving duck flocks in Central Java, Indonesia.

District	Distance per journey (km)		Cumulative distance over 6 months (km)		Duration at each location (days)	
	Mean	Median	Mean	Median	Mean	Median
Pemalang (N)	12	7	44	30	46	29
Batang (N)	21	13	52	25	68	43
Purworjeo (S)	22	19	69	63	57	49
Klaten (S)	42	18	101	100	69	63

**Table 2:** Descriptive statistics on travel distance and time spent per location for each month for moving duck flocks in Central Java, Indonesia.

Start month of journey	N of flocks commencing a journey	Distance travelled (km)		Days at each location	
		Mean	Median	Mean	Median
Dec-08	14	11	9	31	28
Jan-09	14	25	18	57	62
Feb-09	14	30	23	47	29
Mar-09	8	29	19	54	29
Apr-09	19	14	14	62	49
May-09	9	27	13	75	106

The mean cumulative distance travelled per flock over the 6 month monitoring period was 65 km (median 62 km, minimum 13 km, maximum 170 km). Moving duck farmers travelled with an average of two duck flocks during each movement. The median number of female ducks in a flock was 221 and the median number of male ducks was 3 [all flocks had female ducks, and 91% (N = 71) of flocks had male ducks]. For 70 movements (which represent 90% of all movements conducted) the type of transport was specified; 52% (N = 36) of movements were conducted only by vehicle (including cars, trucks, motorcycles and bicycles), 37% (N = 26) of movements combined use of transport vehicles with walking and 11% of movements were conducted (N = 8) by walking only. The median distance travelled by walking was 16 km, while the median distance travelled using any other type of transport was 14 km. Average log-transformed travel distances did not differ between the types of transport (F Statistic = 0.907, df 1 and 68, p = 0.344). A total of 63% (N = 44) of movements were conducted together with other duck flocks; 45% (N = 20) by walking together and 55% (N = 24) by sharing a transport vehicle. The median number of flocks sharing transport was two, with a maximum of six flocks sharing transport (for the latter a truck was used). No disinfection was used on any of the transport vehicles. For 68% (N = 53) of the scavenging locations, farmers reported simultaneous sharing with other duck flocks, for 13% (N = 10) simultaneous sharing occurred infrequently and for 19% (N = 15) no or rare sharing occurred. A total of 40% (N = 31) of scavenging locations were shared with chickens, 32% (N = 25) with wild birds and 28% (N = 22) with Muscovy ducks. At 35% (N = 27) of locations contact with people (apart from the flock owner) was possible.

There was no difference in the number of scavenging locations used over the study period between districts (Kruskal-Wallis χ^2^ = 3.5, df 3, p = 0.320). There was a significant difference between districts in the cumulative distance travelled per flock (Kruskal Wallis χ^2^ = 8.0, df 3, p = 0.04) and between individual journeys by flocks (Kruskal Wallis χ^2^ = 9.8, df 3, p = 0.02). Also, the average number of days spent at a particular location differed between districts (Kruskal Wallis χ^2^ = 8.4, df 3, p = 0.04) (Table 1). There was no significant difference in the use of transport types between the four districts (Pearson χ^2^ = 11.4, df 9, p = 0.253) and between the districts in the north and in the south (Pearson χ^2^ = 3.1, df 3, p = 0.376).

For the use of 73% (N = 57) of scavenging locations no payment was made to rice field owners. However, if a payment was made, usually it was through providing eggs (62% of 21, ranging between 2–15 eggs daily), through both local currency and eggs (24%) or in local currency only (14%).

### AI virus and antibody prevalence

**Fig 3:**
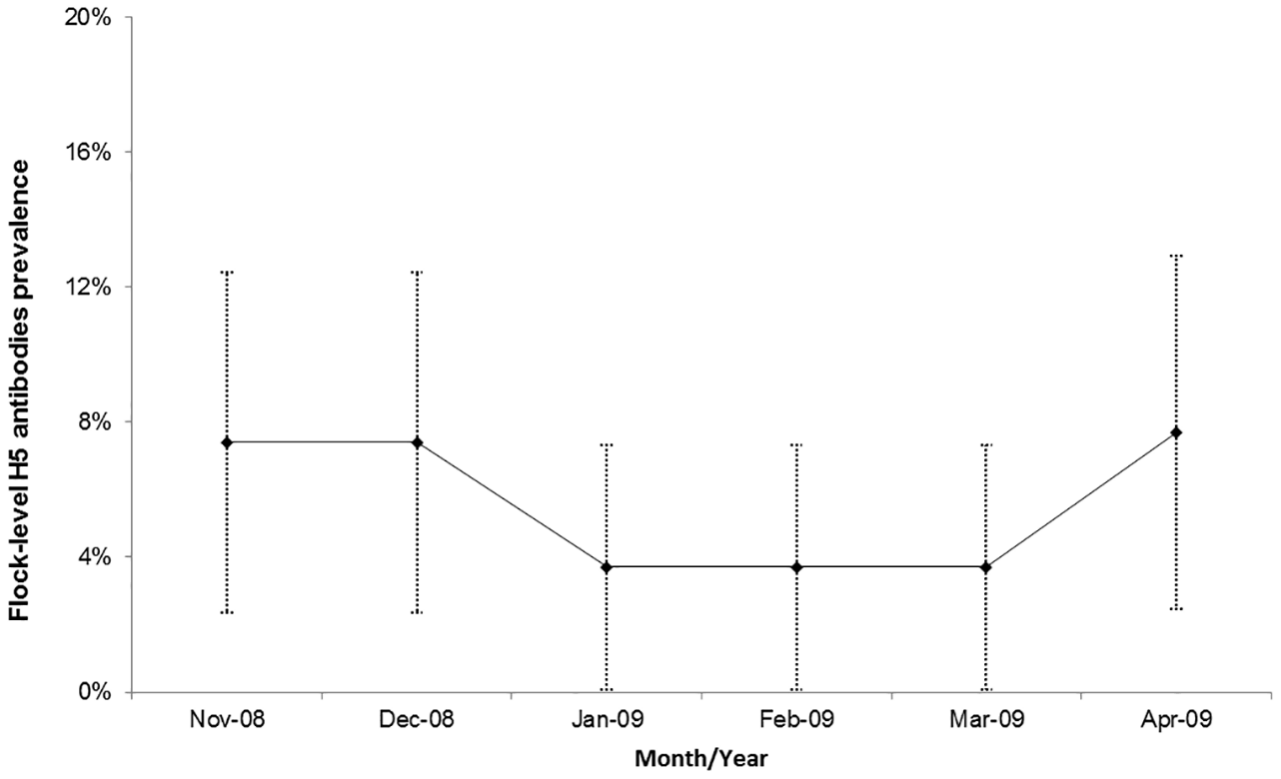
Flock-level prevalence (including 95% confidence intervals) of H5 antibodies of moving duck flocks monitored over a period of 6 months in Central Java, Indonesia.

No HPAI outbreaks were reported from any of the moving duck flocks during the study period. A total of 1608 blood samples were collected from the 27 flocks over the six month study-period and tested for H5 antibodies. None of the ducks were vaccinated against HPAI. The bird-level period prevalence for H5 antibodies was 0.6% (95% CI: 0.4%, 0.8%). The flock-level period prevalence (based on considering a flock to be positive if at least one bird of the 10 sampled per flock tested positive) for H5 antibodies was 5.6% (95% CI: 3.8%, 7.4%) (Fig 3). Two districts (Klaten and Pemalang) had no H5 antibody positive birds during the study period. All cloacal and oropharyngeal swabs that were collected tested negative for H5 viral RNA.

### Network of professionals involved in the moving duck management

A total of 130 professionals (= actors) were identified by the moving duck farmers in the four districts (Fig 1). These included 26 transporters of moving duck flocks, who loaded duck flocks on their transport vehicles and moved them between scavenging locations, 38 hatcheries, who purchased eggs and sold ducklings back to moving duck flock owners and 66 rice paddy owners, who made their rice paddies available to flocks for scavenging.

**Fig 4:**
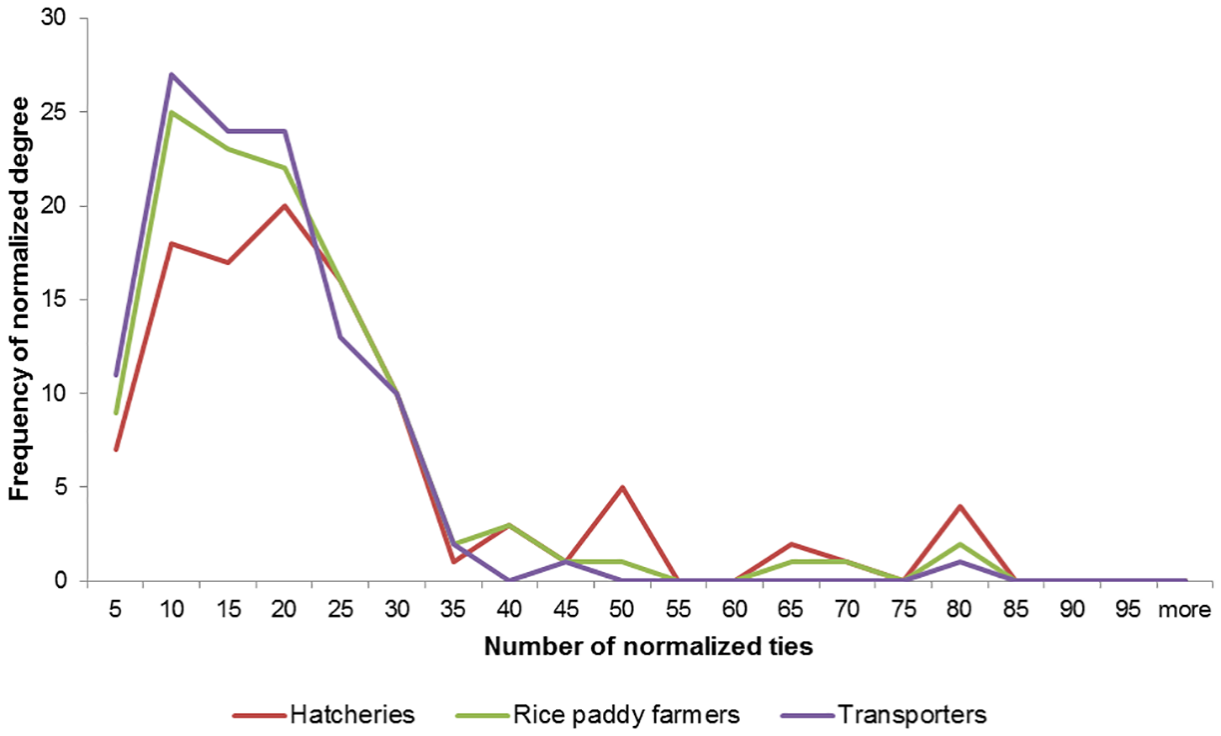
Frequency of normalized degree and number of normalized ties for three categories of actors involved in the moving duck flock production system in Central Java, Indonesia.

The degree distribution in the social network of actors was skewed as would be expected in a natural network, with some actors having a higher degree than others (Fig 4, Table 3). Normalised degree did not significantly differ among the three types of actors (F Statistic = 2.5, df 2,127, p = 0.088). For all four districts the basic reproductive number, R_0_, was greater than 1 (R_0_: Pemalang = 4.03, Batang = 7.31, Klaten, 3.25, Purworejo = 3.07), with the mean R_0_ being 4.41.

**Table 3:** Descriptive statistics of the social networks defined by different categories of actors involved in the moving duck flock production system in four districts of Central Java, Indonesia.

Category		Pemalang	Batang	Klaten	Purworejo
**No. of Actors (Nodes)**	Total	40	19	45	26
	Hatchery	15	7	15	1
	Rice paddy	18	10	20	18
	Transporter	7	2	10	7
**No. of Moving Duck Flock Farmers**		9	9	9	8
**Measures of Centrality**					
Normalized Degree (Freeman)	Mean	15.13	32.75	13.94	11.69
	SD	8.73	11.13	5.66	6.56
	Network centralisation	27.26%	25.49%	11.58%	22.00%
Normalized Betweeness (Freeman)	Mean	0.20	3.30	0.63	0.19
	SD	1.26	6.07	2.40	0.96
Normalized Closeness (Freeman)	Mean	3.34	65.88	3.31	4.64
	SD	0.69	10.96	0.76	0.65
**Network structure**					
Components		7.00	1.00	5.00	7.00
Cutpoints		1.00	0.00	3.00	1.00
Fragmentation		0.77	0.00	0.70	0.84
Density		0.15	0.51	0.14	0.12
Overall clustering coefficient		0.99	0.85	0.97	0.98
Weighted clustering coefficient		0.93	0.77	0.91	0.86
**Basic Reproductive Number**		4.03	7.31	3.25	3.07

**Fig 5:**
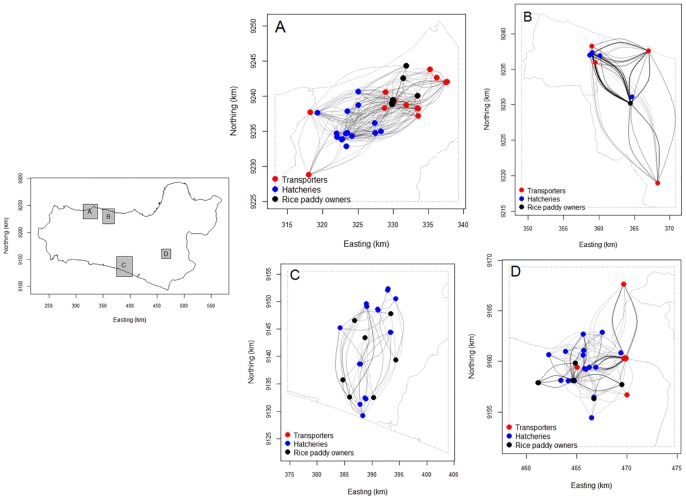
Geo-referenced networks of actors involved in the moving duck flock production systems in four districts of Central Java, Indonesia. Links between actors are based on their interactions with moving duck flock farmers. The map was generated in R 3.0.2. (R Foundation for Statistical Computing) using the igraph package.

**Fig 6:**
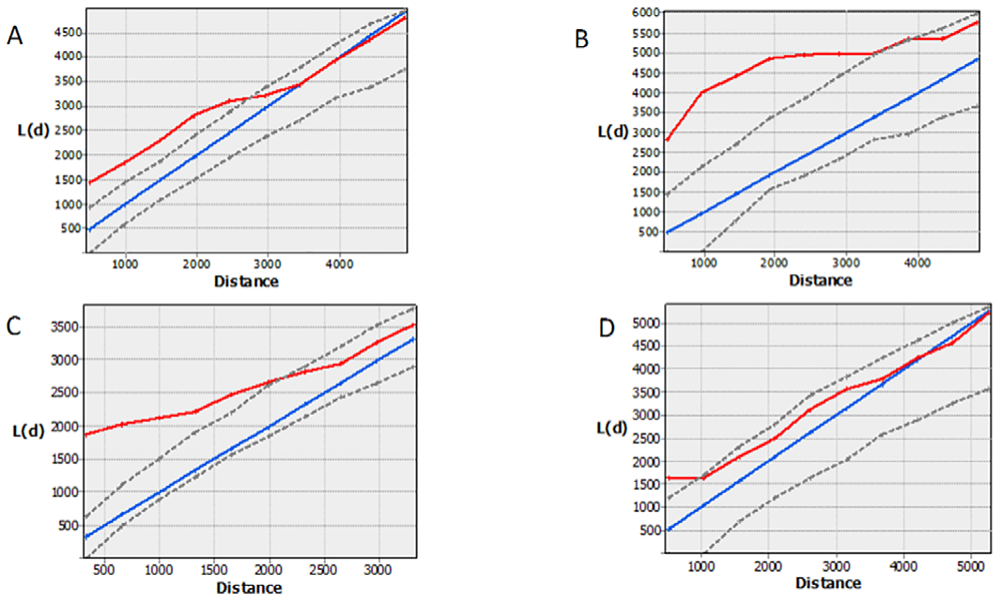
Plots of spatial k functions derived from the spatial distribution of actors involved in the moving duck flock management in four districts of Central Java, Indonesia. A) Pemalang B) Batang C) Klaten D) Purworejo. Blue—expected k-function, Red—observed K-function, Grey– 95% simulated confidence envelope.

The geo-referenced network structure of actors highlights the complexity and intensity of the interactions generated by the activities of the moving duck farmers and the variation in the spatial distance of actors between districts (Fig 5). The K-function as an estimate of the scale of spatial autocorrelation highlighted the clustering of actors. Within all four districts clustering of these actors occurred at distances of less than 3.5 kilometres (Fig 6).

## Discussion

The role of moving duck flocks in parts of South-East Asia is controversial, particularly because they fulfil a useful function within the rice production systems in these countries, including pest control, soil loosening and manure provision, while also maintaining HPAIV H5N1 infection. Although it has been highlighted previously that higher duck density is associated with increased risk of HPAI H5N1 outbreak occurrence, we note that these analyses were based on census data of duck numbers [5, 25]. Based on our experience working with government agencies in Vietnam and Indonesia, no information on moving duck flocks is generally compiled by veterinary authorities and census data only record ‘stationary’ flocks. The plausibility for moving duck flocks to play a role in HPAIV spread is based on potential contact with infected wild birds in rice paddy fields, contact between different moving duck flocks, and the ability to range over large distances during a production cycle. In addition, the fact that these flocks are being moved around makes it difficult for animal health authorities to integrate them into any HPAI control and prevention programmes. A national control strategy where for example certificates to allow movement of the moving flocks are provided was not established in Indonesia at the time of this study. The current study, for the first time describes the moving duck flock production system in Indonesia in quantitative terms. We were also able to highlight two factors specific to moving flock production systems that are likely to play a role in the spread of HPAI virus. These are firstly, the physical movement of ducks by flock owners and secondly, the direct and indirect links between different flocks through interactions with transporters and hatcheries and the use of rice fields.

We were also able to estimate R_0_ for the network of actors involved in the moving duck production system. For all four districts R_0_ was greater than 1, indicating that epidemics in the social network were likely if a moving duck farmer’s flock was infected or the environment or physical structures of an actor were contaminated (e.g. transport vehicles, rice paddies, hatchery equipment) with HPAI H5N1. We used published values for the infectious period and the transmission probability to calculate R_0_ for the moving duck networks.

However, it should be noted that published estimates of HPAI infectious periods vary greatly. For example, the mean infectious period for HPAIV infection in turkeys was estimated to be 1.47 days, but turkeys, similar to chickens, are likely to succumb rapidly to the disease [26]. We are confident that published median infectious period of 5 days for HPAIV infection in ducks was adequate for the moving duck network analysed here [23]. For the per-contact probability of virus transmission we used values that were derived from data collected during the 2003 HPAI H7N7 epidemic in the Netherlands [24]. Using a maximum likelihood estimation approach the authors calculated probabilities of virus transmission conditional on the contact originating from an infectious farm. For example, the transmission probabilities was 0.0414 per feed delivery contact, 0.308 per egg transport contact, 0.133 per other-professional contact, and 0.246 per rendering contact. The authors indicated that the lower per-contact probability of infection per feed delivery compared to egg transport may be due to the difference in degree of contact: unlike egg pick-ups where the eggs have to be collected from the egg room, feed delivery may not involve accessing storage rooms or poultry houses. We considered that the per-contact probability for feed-deliveries was appropriate for our study in which vehicles were used to transport ducks between locations without entering poultry confinement areas. Similar probabilities of HPAI H5N1 infection were estimated for egg collectors that had visited small-scale broiler chicken farms with H5N1 outbreaks in Indonesia (probabilities of infection 0.032–0.013 for collectors using a truck and 0.021–0.086 for collectors using a pick-up) [27]. Nevertheless we used a conservative estimate for the probability of virus transmission and derived relatively large estimates of R_0_, which highlights the strong interconnectedness of the actors involved in duck management network.

Actors are likely to be unaware of the direct and indirect connections they share with actors of other categories (as these actors are only linked by interacting with the same moving duck flock), which is likely to increase the risk of HPAIV transmission. We propose that actors should be made aware of the potential role they play in transferring HPAIV from infected to susceptible poultry flocks via educational programmes aimed at risk mitigation. This also highlights the role that improved biosecurity between contacts and/or a reduction in contact frequency can play in eliminating between-actor and between-farm spread of HPAIV during future epidemics.

The moving duck flock population monitored in this study were layer ducks, representing ducks which are kept for a longer production cycle compared to meat ducks. Layer ducks might possibly more likely to be exposed to LPAI H5 and/or past HPAI virus because of this longer production period and hence we should be cautious when extrapolating our findings beyond this type of moving duck flock system. However, all moving duck flocks identified for this study were layer ducks highlighting that layer ducks are the most common moving duck production system in the study area.

No HPAI outbreaks occurred and no HPAIV RNA was detected in cloacal and oropharyngeal swabs during the study period. However, the risk of HPAIV transmission is imminent throughout the year and varying HPAIV transmission risk has been associated with seasonal rice harvest peaks [5].

The initial proposed banning of live bird markets to reduce the risk of HPAIV transmission (e.g. Egypt, Vietnam, China) [28–29] was difficult to implement and was ethically questionable given that the livelihood of small scale farmers in these countries is dependent on these activities. Nevertheless, during HPAI outbreaks market closures were implemented successively to stop the spread of the virus, but recent research has highlighted that rest days are sufficient in reducing market infectivity [30]. This might also be applicable to the moving duck production system—instead of banning the movement of duck flocks, several rest days plus disinfection of vehicles used for transporting ducks and of rice paddies used for duck scavenging might be appropriate. The rationale here is that survival of HPAIV H5N1 in the environment is limited, for example less than 1 day in mud, up to 4 days in rain water [31] and up to 3 days in paddy field water at 25–32°C [32] (although the salinity of the water and possibly the levels of exposure to UV radiation may also play a role [33–34]).

The network actors identified in this study are known to be present in small to medium duck management production systems in other duck producing countries in the region, such as Vietnam [8] and Thailand [35], although detailed field information on the duck movements in these countries still need to be generated.

In summary, we propose that HPAIV transmission is influenced by the short and long distance movement of duck flocks. Contacts between duck flocks provide opportunities for localised spread of HPAI virus while the intensive professional network of actors involved in the duck management (e.g. transporter or hatchery owners) might support the long-distance spread of the virus. Overall, our study highlights the important role of humans and their activities related to the management of moving duck flocks in the dissemination and the mechanical transmission of HPAIV.

## Supporting Information

S1 File Questionnaire used to interview moving duck farmers. (PDF)
