# Supplementary material for: Who Is Spreading Avian Influenza in the Moving Duck Flock Farming Network of Indonesia?
Source: PLoS One. 2016 Mar 28;11(3):e0152123. doi: 10.1371/journal.pone.0152123 (PMC4809517; doi:10.1371/journal.pone.0152123)
Supplement: S1 File — (PDF) [file pone.0152123.s001.pdf]

MOVING DUCKS IN CENTRAL JAVA INDONESIA

MONTHLY QUESTIONNAIRE

Farm reference number:

Date of interview:

Current location of moving duck flock: village, sub-district and province	
Current location of moving duck flock: GPS readings	S:
	E:

Information on moving duck flock structure, movements, scavenging and sales

1. How many moving duck flocks do you have **TODAY**? *For each flock outline the number of ducks, the breed, the age, the vaccination status and the proposed date of AI vaccination. M=Male, F=Female*

Duck group number	Number	Breed	Age today	AI vac already		Date of AI vaccination (first)	Date of AI vaccination (booster)	Location where AI vaccinated
				Yes	No			
**1** M				□	□			
**1** F				□	□			
**2** M				□	□			
**2** F				□	□			
**3** M				□	□			
**3** F				□	□			

2. Use the calendar provided to check/specify the location of the moving duck flock on a daily basis since the last sampling. *Use a* ***location reference letter*** *in the calendar and below. Ensure all dates are filled in. List details for each location reference letter below.*

Location reference letter	From which date	Until which date	District	Sub-district	Village	Sub-village	Reason for being at this location	List name of rice paddy owner
A								
B								
C								
D								
E								
F								

3. Indicate all locations of the moving duck flock since the last sampling on the map. *Use the same* ***location reference letter*** *above.* ***Please tick the box when completed.*** □

Comments by farmer on any of these questions:

4. Specify the type of transport you used to move the ducks to each location.

Location ref. letter	Type of transport	Vehicle disinfection before transport			N of people involved in transport	Transport shared with other flocks. Specify N flocks.	Name of transport owner
		Yes	No	Don't know			
A		□	□	□		□ ……………..	
B		□	□	□		□ ……………..	
C		□	□	□		□ ……………..	
D		□	□	□		□ ……………..	
E		□	□	□		□ ……………..	
F		□	□	□		□ ……………..	

5. Specify the type of scavenging areas used and the number of duck flocks sharing each scavenging location.

Location ref. letter	Rice fields	River banks	Crops of other kind (specify)	N of duck flocks sharing this area	
				From your group	Other duck flocks
A	□	□	□ ……………….		
B	□	□	□ ……………….		
C	□	□	□ ……………….		
D	□	□	□ ……………….		
E	□	□	□ ……………….		
F	□	□	□ ……………….		

6. What arrangements do you have with the rice field owner to have your birds scavenging there? Please indicate how much money you have to pay for what period of scavenging (per month, per 2 months etc.) and for what size of the scavenging area (in ha).

7. Specify the type, amount and the source of additional feed used and the type of confinements (material, size of area etc.) used at each location.

Location ref. letter	Additional feed			Type of confinement	
	Type of feed	Amount given per day	When and where obtained	Day	Night
A					
B					
C					
D					
E					
F					

8. Did your moving ducks share any scavenging areas or had contact any other animals or people since the last sampling? *Please indicate the frequency and the when the contact occured.*

	Often	Some times	Seldom	Never	Please indicate what time of day or night
Other ducks	□	□	□	□	
Chickens	□	□	□	□	
Geese	□	□	□	□	
Wild birds (specify) …………	□	□	□	□	
Other poultry (specify) ………….	□	□	□	□	
Dogs	□	□	□	□	
Cats	□	□	□	□	
Water buffalos	□	□	□	□	
Other animals (specify) ………..					
People other than those working with the duck flock	□	□	□	□	

Comments by farmer on any of these questions:

9. When are your ducks scavenging **today**? *Indicate details for each group and scavenging session.*

Duck group number	Session	Start time	Finish time	Type of supervision				
				When moving to area	While scavenging	When returning from area	No super-vision	Other (specify)
1	1			□	□	□	□	□ …………
	2			□	□	□	□	□ …………
2	1			□	□	□	□	□ …………
	2			□	□	□	□	□ …………
3	1			□	□	□	□	□ …………
	2			□	□	□	□	□ …………

10. Were any ducks sold or given away since the last sampling? *If yes, give the details below.*

Date	Number		Age	To whom?	Location to where sold or given away	Transport to sale/given away location	Sale price per duck
	sold	given away					

11. Did you try to sell (or give away) ducks, but returned with unsold ducks? Yes No

If yes, give details below, i.e. indicate how many, why and what was done with these birds.

Information on egg production and hatching

12. How many eggs were laid since the last sampling? *For duck group refer to question 1.*

Duck group number (Q1)	Number of eggs laid
1	
2	
3	

13. Did you lose eggs to different causes since the last sampling?

Yes No

If yes, please indicate causes and the number of eggs lost by each cause below.

Predators	Theft	Other (specify)	Unknown
…………….	………………..	…………………….	…………………….

14. Have you observed poor egg shell quality since the last sampling?

Yes No

15. Were any eggs sold or given away since the last sampling? *If yes, give the details below.*

Date	Number		Location to where sold or given away	Transport to sale/given away location	Sale price per egg
	sold	given away			

16. Did you try to sell (or give away) eggs, but returned with unsold eggs? Yes No

If yes, give details below i.e. indicate how many, why and what was done with these unsold eggs.

Comments by farmer on any of these questions:

17. Since the last sampling did you had eggs incubating and did you had any hatchings? *If yes, please give details below.*

Location *(details and if home)*	Species and breed	Number incubating	Method of incubation	Source of fertile eggs		Details on incubator	Specify N hatched and what was done with the hatched ducklings?
				Home laid	Bought		
				□	□		
				□	□		

Information on purchases

18. Were ducks purchased or given to you since the last sampling? P*lease give details below.*

Date	Breed	Number		Age when obtained	From whom?	Location obtained	Transport to flock	Price per duck	HPAI vaccinated		
		bought	given						Yes	No	Don't know
									□	□	□
									□	□	□
									□	□	□
									□	□	□
									□	□	□
									□	□	□

19. How were new purchased or given ducks incorporated into the flocks?

Separate initially (specify how long and where)	Mix immediately	Other (specify)
□…………………………………………….	□	□…………………………….

20. Were any eggs purchased or given to you since the last sampling? *If yes give the details below.*

Date	Number		Location obtained	Transport to farm	Price per egg	Purpose of eggs	
	bought	given				eating	hatching

Information on duck health

21. Did you have any ducks deaths in your moving duck flock since the last sampling? *If yes, give details in the table below. For duck group refer to question 1.*

Date	From which duck group (Q1)	Age at death	Number dying	Symptoms observed

22. What was done with the carcasses of the dead ducks? *Tick one or more boxes of the following.*

Burned		Buried	
Sold		Eaten	
Processed for fish feed		Thrown away	
Nothing-just left there		Other (specify)……….…...	

23. Did you have any ducks with sickness but not death in your flocks since the last sampling? *If yes, please give details below.*

Date	Number of ducks	Age	Symptoms observed

Comments by farmer on any of these questions:

24. How were these sick ducks treated? *Specify date from question 23 and provide details.*

Date (from Q 23)	Separated from flock		Medications given	
	Yes	No	Type of medication	Route of administration

25. Did you have your moving ducks vaccinated against any of the diseases specified below? *If yes, please provide details on the vaccination.*

Vaccine	Vaccinated	Date of vaccination	Location where vaccinated	Person administrating vaccine
Duck plague	□			
Pasteurella	□			
Other (specify) …………………..	□			

26. Have you seen any dead birds along the way? □ Yes □ No

If yes, please provide details on the dead birds (location, type of birds, number of dead birds, breed of dead birds etc.).

Information on people with contact to ducks

27. How many people or workers are currently working with the moving ducks including you?

28. Did any of these people or workers visit villages or towns during the journey? *If yes, give details on reason for visiting, and what happens with the ducks at this time.*

Village and sub-village	Reason for visiting	Duck management during visit

29. Did any of these people or workers visit any other places since the sampling? *Tick one or more boxes of the following.*

Other duck flocks	Other chicken flocks	Markets	Cock fights	Other (specify)	Don't know
				………………….	

30. Did anyone else visit your moving flock since the sampling? *Tick one box per row. If you ticked yes specify the frequency and details of this visit.*

	Yes	No	Don't know	Frequency and details on visits
DINAS person/veterinarian				
Poultry trader				
Egg trader				
Manure trader				
Poultry farmers				
Other farmers (specify) ………………….				
Delivery person				
Other (specify) …………………….				

31. Did you keep a dog with your moving duck flock? □ Yes □ No

Comments by farmer on any of these questions:
